# New Frontier in Glycoprotein Hormones and Their Receptors Structure–Function

**DOI:** 10.3389/fendo.2015.00155

**Published:** 2015-10-19

**Authors:** Mariusz W. Szkudlinski

**Affiliations:** ^1^Trophogen Inc., Rockville, MD, USA

**Keywords:** glycoprotein hormone, glycoprotein hormone receptor, structure–function, protein engineering, charge cluster, super–agonist, biosuperior, biobetter

## Abstract

Last two decades of structure–function studies performed in numerous laboratories provided substantial progress in understanding basic science, physiological, pathophysiological, pharmacological, and comparative aspects of glycoprotein hormones (GPHs) and their cognate receptors. Multiple concepts and models developed based on experimental data in the past stood the test of time and have been, at least in part, confirmed and/or remained compatible with the new structures resolved at the atomic level. Major advances in understanding of the ligand–receptor relationships are heralding the dawn of a new era for GPHs and their receptors, although many basic questions still remain unanswered. This article examines retrospectively several basic science aspects of GPH super-agonists and related “biosuperiors” in a broader context of the advances in the ligand–receptor structure–function relationships and new mechanistic models generated based on the structure elucidation. Due to selective focus of my comments and perspectives in certain parts, the reader is directed to the most relevant publications and reviews in the field for more comprehensive analyses.

## Origins and Evolution of Function

The family of glycoprotein hormones (GPHs) consists of luteinizing hormone (LH), chorionic gonadotropin (CG), follicle-stimulating hormone (FSH), and thyroid-stimulating hormone (TSH), which are heterodimers formed by the non-covalent association of a common alpha (α) and a hormone-specific beta (β) subunit. Structurally, GPHs and their subunit ancestors belong to the cysteine-knot growth factor superfamily and due to relatively high glycosylation are recognized as the most complex protein hormone molecules (1–3). Their cognate GPH receptors (GPHRs) are type A leucine-rich repeat (LRR)-containing G-protein-coupled receptors (LGR) with a large glycoprotein extracellular domain (ECD). Early ancestors of GPHs and their receptors emerged at the origin of metazoan animals (multicellular mitochondrial eukaryotes) (4), although two domains of GPHRs, LRRs and 7-helix transmembrane domain (TMD), have much earlier evolutionary origin and are very well-diversified in extremely large number of functionally unrelated proteins in animals and plants (5). Parallel evolution of GPHs, their subunits, and cognate GPHRs was previously studied and discussed in detail (2, 6, 7). An evolution of the receptor–ligand interface likely progressed through the series of fine-tuning within the concave face of the LRRs and activating configuration within the hinge region located between the LRRs and TMD. Interestingly, as previously proposed (6), numerous early vertebrate GPHRs are functioning at least in part by utilizing their constitutive activity, which is determined in each individual cell by the number of receptors expressed in its cell membrane. Remarkably, nematode LGRs are constitutively activating only the G_s_/cAMP, but not G_q_/IP_3_ inositol phosphate pathway (8). In addition, comparative analysis of GPHRs signaling may suggest that G_s_/cAMP pathway as the only mechanism of the agonist-dependent and -independent receptor activation has evolved into more diversified and complex signaling system (9, 10).

Significant level of inherent constitutive activity is present in various vertebrate GPHRs (11–13), including wild-type (WT) hTSHR. In sharp contrast, human LHRs and FSHRs are activated almost exclusively by their respective ligands and the number of natural or artificial receptor mutations in their respective ECDs causing constitutive activity is very low (14–16). With regard to the TSHR, there is an apparent correlation between a high level of basal constitutive activity and much more relaxed ligand specificity (promiscuity), which is exemplified by the prevalence of hCG-induced subclinical or overt hyperthyroidism in the first trimester of pregnancy (17).

## Charge Cluster in the Common α-Subunit

**Figure 1:**
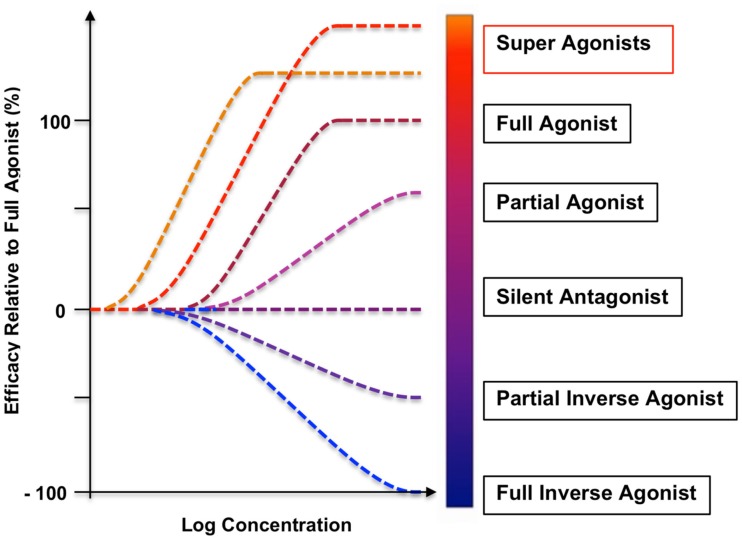
**A range of dose–response curves and relative efficacy spectrum of GPHR ligands**. The efficacies of the selected classes of ligands are illustrated by the *in vitro* stimulation of cAMP production and comparison with an endogenous, WT agonist (full agonist with 100% intrinsic efficacy). Although the term “super-agonist” has not been yet officially addressed in the NC-IUPHAR nomenclature, super-agonists show higher efficacy than full agonists, variable assay-dependent increases in receptor-binding affinity and potency, differences dependent on the receptor densities, differences related to the degree of signal amplification in the activation cascade, and significant enhancement of clinical efficacy in human and veterinary applications. High affinity super-agonists are especially desirable in various disorders with largely impaired receptor-binding and cell-surface expression (22, 24, 31). Multiple partial agonists of all GPHRs and TSHR-blocking anti-TSHR antibodies (silent or orthosteric antagonists) have been studied (32), but only one human monoclonal anti-TSHR antibody was recognized as an inverse agonist with a significant suppression of the basal constitutive activity of the WT TSHR (33).

Significant contribution of electrostatic interactions to high affinity receptor binding has been recognized for various ligand–receptor pairs, including different cysteine-knot growth factors and their respective receptors (18). Accordingly, a long-standing postulate held that charge–charge interactions are of major importance in the TSH–TSHR interactions (19). For the entire G-protein-coupled receptor, strong statistical evidence was provided that negatively charged amino acids are enriched in the ECDs, including extracellular loops (ECLs), but positively charged amino acids dominate within the intracellular domains (20). Design and sequential development of human TSH and gonadotropin super-agonists (Figure 1) were described previously in details (1, 21, 22). Our early mutagenesis studies, which has been recognized as “the advent of super hormone drugs” (21, 23) focused primarily on the 11–20 region of the human α-subunit. These studies have revealed that a basic charge cluster in this region, which has evolved in vertebrates and disappeared in apes and humans, is an important modulator of hormone–receptor binding and activation. Amino acid substitutions to positively charged lysine (K) or arginine (R) in the 11–20 region individually (T11K/R, Q13K/R, E14K/R, P16K/R, Q20K/R) and in various combinations increased the potency and efficacy of hTSH and hCG (21). Such human analogs remain highly specific for their respective receptors and inactive (up to 1000-fold higher concentration) at the other GPHRs (24). The effect of these substitutions on the *in vitro* bioactivity was highly correlated with their effects on the receptor binding activity. It was repeatedly demonstrated in media and buffers with various salt concentrations, and confirmed by studies in other laboratories (25, 26) as well as by using CHO-TSHR cells with largely depleted pool of the negatively charged cell surface proteoglycans. Notably, mutations to alanine did not alter hormone activity, indicating that only selective substitutions to K or R amino acid residues are causing an enhancement of cAMP and IP_3_ production, iodine uptake, proliferation of FRTL-5 cells, thyroxine and progesterone production, respectively (1, 21, 24). All our previous theoretical models of GPH–GPHR interaction derived from super-agonist studies were placing the mutagenized α-subunit αL1 and αL3 loops in a close proximity to the hinge region and the ECLs of the receptor TMD (1, 27), very similar to early structural predictions by Jiang et al. (28) and highly compatible with two epitope-mapping studies (29, 30).

**Figure 2:**
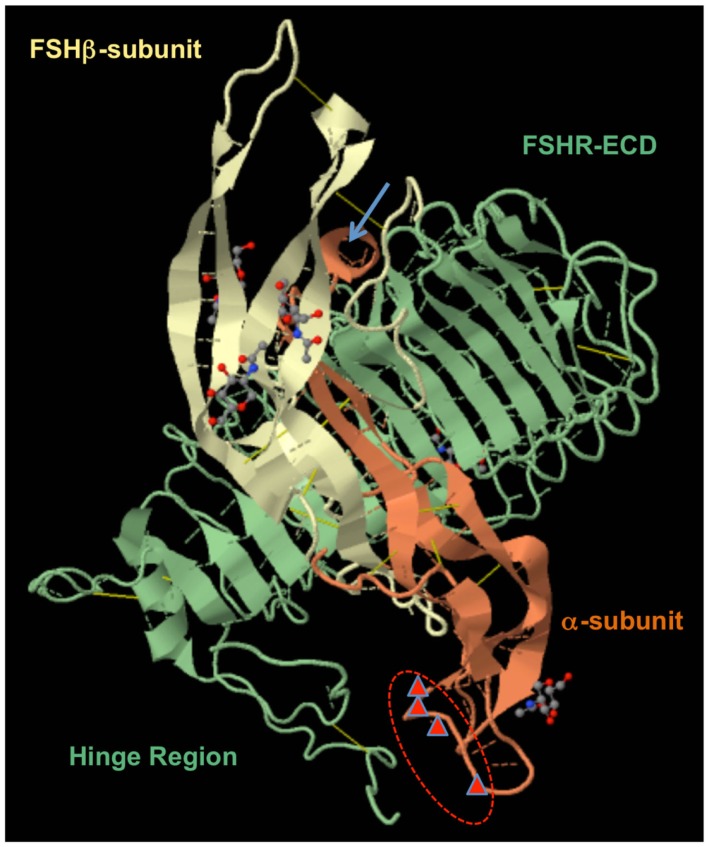
**FSH–FSHR/ECD complex (PDB 4AY9) as reported by Jiang et al. (35)**. The locations of human α-subunit αL1 residues 13, 14, 16, and 20 are marked by red triangles and circled in red. The α-subunit α-helix is seen at the top as a light brown circle and is marked with a blue arrow. LRRs together with the hinge region are forming one large domain interconnected with three disulfide bridges. The recent “two-step model” subdivided this domain into the hormone-binding subdomain (HBSD) and signal-specificity subdomain (SSSD) (36).

Super-agonists of human GPHs, also named as “superactive analogs,” have been generated by introduction of positively charged amino acid residues in selected locations of the αL1, αL3, and βL3 beta-hairpin loops (1, 21, 27). Our “signal-enhancing αL1 loop mutations” were described as fully consistent with the model of receptor activation based on the structure of FSH bound to N-terminal cysteine cluster together with the LRRs proposed in 2005 (34). Highly unique position of the α-subunit αL1 13–20 mutations was more recently confirmed by Jiang et al. and the structure of hFSH bound to the entire ECD of hFSHR (35) (Figure 2). Moreover, Jiang et al. (36) discovered that such α-subunit αL1 loop amino acid substitutions to K (21) or R (37) are “concentrated near the top right side of the ‘activation pocket’ generating stronger electrostatic interaction to pull and lift the sulfated-tyrosine 335 (sTyr335) of the FSHR hairpin loop,” which is essential in the receptor activation.

## GPH Super-Agonists – Tools in Structure–Function Studies

Two step activation mechanism proposed based on FSH–FSHR/ECD complexes (35, 36), and other structure-based models (38, 39) incorporated and explained, at least in part, several our previous findings as described below.

First, the new structure-based model by Jiang et al. (36) is placing much emphasis on the signal-specificity subdomain (SSSD). It explains, at least in part, why positive charge cluster in the αL1 loop, from our studies initiated in 1995, was rescuing (restoring) several major “loss-of-function” mutations including two mutations of K51 in the α-subunit (K51A and K51P) (40), which later have been found to be essential in the formation of salt bridge with highly conserved D153 within the receptor LRRs domain (34) and several others described elsewhere (1, 41) [see also Ref. (3, 38, 42, 43)]. Moreover, largely reduced binding activity and potency of single-chain hCG and its minimized variants were restored using αL1 loop substitutions (α4K and α4R) (25, 26). Remarkably, also the LH activity of the hTSH/hCG “seat-belt” “determinant loop” chimera was further increased by concomitant introduction of a cluster of K residues (α4K) into a highly distant from “seat-belt” αL1 13–20 domain (41). The results of several other published and unpublished studies further reinforced the role of αL1 loop basic charge cluster in the compensatory mechanism functioning within the SSSD.

Second, activation of the TSHR by free or fused homodimeric αL1 α-subunit analogs, but not the WT human α-subunits has been detected in a concentration range only 1–2 log orders higher than that of hTSH-WT (44, 45). Such agonistic activity of α-subunit analogs was observed only in free, non-tethered forms, but not in the yoked subunit-TSHR complexes constrained by the fusion of α-subunit and TSHR. These findings first challenged the most dominant at that time concept that the hormone-induced receptor activation is highly restricted to interactions primarily or exclusively within LRRs. In the light of new structures, the location and configuration of “signal-enhancing substitutions” in the activating complex is important and may not be altered, particularly considering much weaker binding of the free α-subunit analog than the hormone heterodimer. This explanation is also consistent with our data indicating that synthetic linear and cyclic peptides corresponding to the human α-subunit 11–20 residues with α4K substitutions and 10 μM concentrations are not inducing any significant cAMP responses (45).

Third, testing hTSH-WT in comparison to hTSH super-agonist TR1401 with the same α4K substitutions using mutated hTSHR in the hinge region, revealed that non-conservative substitutions E297, E303, and D382 are strongly reducing TR1401 binding and cAMP signaling based on TSHR cell surface expression normalization using FACS analysis (46). Two substitutions to the positively charged K residue (E297K and D382K) led to particularly strong decrease of TR1401 binding and cAMP signaling. Regardless of specific mechanism of electrostatic steering and repulsion involved, and considering well known limitations of simultaneous hormone and receptor mutagenesis (47–49), such studies indicated that the analogs with a limited number of “gain-of activity” substitutions can serve as an excellent tool for probing hormone–receptor interactions. Use of such minimally mutagenized human hTSH analogs (e.g., TR1401 with α4K) together with largely different bovine bTSH-WT (36 amino acid difference) combined with systematic receptor mutagenesis and TSHR expression normalization allowed to narrow interactions areas within the hinge region and SSSD (49, 50).

Fourth, heterothyrotropic activity of mammalian GPHs can be increased in goldfish by the same α4K substitutions and interactions within the SSSD and hinge region. Such interactions likely evolved long before diversification of LHR and TSHR receptors (51). Co-evolution of early GPHs and their receptors, driven by positive selection within the hormone-binding subdomain (HBSD), related to the need for adaptation of new functions, likely controlled “spillover” of hormone activity to other GPHRs.

Fifth, in addition to supergonists based on selective introduction of positively charged amino acid residues into the αL1 loop, additional substitutions to R or K in the αL3 and βL3 loops led to noticeable increases in the receptor binding both individually and in combination (21, 52–54). The oligomerization of GPHRs was observed in the past, but recent structures provided more convincing evidence for functional relevance of receptors oligomers, dimers, and trimers. An interaction of βL3 loop with three mutations to R (β3R) located in proximity to the FSHR trimer exosite, suggests potential role of such exosites for additional hTSH βL3 loop binding in trimeric receptors (36, 52, 55), which could be affected by the proportion of receptor trimers in different cells and *in vitro* bioassay conditions. Although, the trimeric FSH model does not precisely predicts the extend of cAMP increase due to signal amplification by the adenylate cyclase, it places again the location of both αL1 and αL3 loops near the TMD and predicts that upon dissociation of trimers into monomers both binding and signaling activities of glycosylated WT hFSH should increase threefold. Dual FSHR signaling by monomers and/or by trimers may serve as a part of evolutionarily based protection of reproductive functions, and constitute a possible compensatory mechanism preserving some minimum level of FSH induced signaling in various stress conditions affecting GnRH pulsatile secretion depressing pituitary hFSH production and secretion, observed during chronic malnutrition or starvation (56, 57). hFSHR and likely hLHR signaling by monomers or trimers may also modulate action of elevated endogenous gonadotropins during malignant cell transformation in the menopausal women and their ovarian epithelial and granulosa cells, later associated with the decreased FSHR expression, low receptor number in the cell membrane as well as altered receptor occupancy, trafficking, and biased signaling (58, 59).

Although, activation of GPHRs induces the coupling of different G proteins (60), most of physiological activities are mediated through a G_αs_ protein induced adenyl cyclase catalyzing the conversion of ATP into cAMP (1). However, as suggested by studies on TSHR deletions and others focusing on GPHRs signaling and trafficking, “biased agonism,” also referred to as “ligand directed signaling” is likely caused by a spectrum of different ligand–receptor complex conformations in combination with other cell-specific factors (1, 61–63). Recent studies indicated that different GPH glycoforms may have distinct effects on signaling and result in a biased agonism (64). Thus, it is expected that each GPH variant may have different and sometimes completely unique signaling pattern. However, there is also pharmacologically justified possibility that super-agonists are in general less capable of inducing multiple conformations and therefore much less biased (65). Selected super-agonists are known to have an extended receptor-residence time, which in turn may affect GPHR interactions with the cell adapter proteins, endosome signaling, and signal compartmentalization (61, 66–68).

In summary, new investigative strategies including “charge scanning and reversal mutagenesis,” “loss-of-function restoration by superagonist,” as well as “loss-of-superagonism” with mutated or truncated GPHRs constitute highly valuable tools in the structure-function studies both in the absence and presence of structural information (1, 46, 53, 69). We have first showed directly using large deletions that the ECD suppresses an inherent constitutive activity of the TMD of the human TSHR (14). Such an intrinsic property of the ECD acting on the TMD as a partial inverse agonist was recognized after introduction of the “driver hemagglutinin (HA) tag-sequence” at the N-terminus of the truncated constructs designed to improve and assess cell surface expression (14). We have demonstrated that the presence of such “driver sequence” in the TSHR and other GPHRs constructs with large ECD deletions in the ECD is absolutely necessary for an efficient cell surface expression (1, 14, 70). Similar “driver sequences” were subsequently used to express and normalize expression of various other GPHR constructs with several major ECD deletions, assess their interactions with analogs, and determine their constitutive activities in both cAMP and IP_3_ signaling pathways (1, 15, 62). Several newer studies analyzing the role of charged residues in the receptor hinge region attributed an inherent agonistic property to this domain and supported our early concept that the receptor charge clusters adjacent to the TMD are critical in a downstream signal transmission (49, 50, 71).

## Development of GPH Biosuperiors and Other Related Molecules

“Biosuperiors” (biobetters or next-generation biological therapeutics) are defined as the second-generation products with substantial advantages over the originator molecules. Biosuperiors have the same receptor target and general mechanism of action as previously approved WT recombinant molecules but include structural changes and/or altered properties that result in an improvement in their clinical profile. GPH biosuperiors can be classified into three main categories: super-agonists, long-acting analogs, and WT molecules with optimized glycosylation, formulation, or delivery.

Long-acting bovine FSH super-agonists with much higher efficacy than all other products in veterinary markets have been developed at Trophogen Inc. and entered clinical trials for superovulation in cows and heifers (72). Human TSH and FSH super-agonists for diagnostic and therapeutic applications in the thyroid cancer of follicular origin and the treatment of infertility, including poor responders in the controlled ovarian stimulation (COS), respectively, have entered the late-phases of preclinical development (22, 24, 37, 73). Long-acting analog of WT hFSH (corifollitropin alfa; Elonva^R^, Merck) has been approved in Europe for COS. It was constructed by fusion of the carboxy-terminal peptide of the β-subunit of hCG to the β-subunit of hFSH. Such additional 28 amino acid residues peptide containing 4-5 *O*-linked carbohydrate chains resulted in twofold increase in the FSH plasma half-life. Elonva^®^ substituted for 7 days daily injection of the WT hormone in the standard COS procedure (74–76). Additional post-approval assessments are expected to determine its clinical convenience value and acceptance in the IVF market. Glyco-optimized highly sialylated WT FSH (FSH-GEX™) developed by Glycotope GmbH is based on its production in the human GT-5s cell line providing more optimal glycan structure pattern than standard CHO cell line (77). Similar efforts are now also directed to engineering CHO cells and generation of more homogenous FSH glycosylation, including “human-like” α2,6-terminal sialic acid linkages (78). Recent developments of various GPH biosimilars, improved formulations (79), injection frequency and convenience-focused preparations have not addressed several largely unmet needs for much higher efficacy-based biosuperiors (80, 81).

Clinical utility of thyrostimulin, GPH-related protein found in both vertebrates and invertebrates (82), still awaits full elucidation and rigorous assessment of specificity, selectivity and potential, if any, therapeutic benefits. Small molecules given orally may also lack sufficient specificity, but modified and/or minimized protein variants may provide sufficient balance between specificity, selectivity, half-life and convenience of enteral or topical administration (83). In contrast to large GPHs, there is largely incorrect perception that highly improved affinity of small molecules to GPHRs should result in parallel increase of their specificity. However, the specificity of small molecules is more relative to the degree of non-relevant binding than the strength of their interaction with specific receptor (84). These reservations aside, during the last 10 years, several new advances have been made in the development of GPHR small molecule ligands and allosteric modulators (85–87).

## Closing Remarks and Future Perspectives

Glycoprotein hormone–glycoprotein hormone receptor structure–function research is evolving into new highly promising phase, which will likely culminate in elucidation of the entire active and inactive structure(s) of hormone–receptor complexes, including constitutively active receptors, entire receptors bound to super-agonists, antibodies and small molecule ligands. The advent of new optical techniques based on FRET and BRET sensors, as well as single-molecule microscopy, will allow more detailed analysis of real-time receptor activation and direct spatial assessment of signaling in the living cells. Such new optical techniques made already possible detection of TSHR signaling to cAMP after receptor internalization into endosomes (68, 88).

In analogy to human genome sequencing, full benefits of the structure–function achievements may not immediately translate into the new drugs and the third generation of GPH biosuperiors and theranostics. Further progress in the understanding of functional and therapeutic potential of signaling bias, receptor trimerization, trafficking, compartmentalization of signaling as well as detailed elucidation of the mechanism of super-agonists binding and signaling, should move this field to a new very exciting times of personalized drugs with predefined pharmacodynamics (PD), pharmacokinetics (PK), and signaling profiles (89).

Future third-generation recombinant protein biosuperiors will likely have even more advantages related to efficacy, potency and half-life, but also in relation to largely improved stability, formulation, bioavailability, and new methods of administration, eliminating the need for multiple injections (83, 90). New automated single-use sensor-based manufacturing technology platforms of biologics as well as largely improved purification and characterization methods should make a whole development process faster, safer, and more efficient, assuming necessary improvement and streamlining in the regulatory agencies, their flexibility, commitment to a case-by-case considerations and willingness to accept well justified unorthodox development strategies.

It is apparent to many biotechnology experts and market analysts that major biosuperiors, which are largely improved versions of the originator molecules, will be the next big opportunity in the entire field of biologics and GPCR protein ligands (80). It is predicted that biotech and pharma companies, well known for innovation and experience with the first-generation recombinant proteins and biosimilars, will be the best positioned to achieve early success with biosuperiors as well as with the biosuperior-based targeting conjugates and the nanoparticles with theranostic capabilities. New highly exciting frontier of precision medicine combining targeted and personalized interventions is already looming on the horizon. The sense of wonder, excitement, and anticipation of the future progress in the structure–function and novel drug design can be well expressed by Carl Sagan’s visionary quote: “Somewhere, something incredible is waiting to be known.”

## Conflict of Interest Statement

The author is Executive Vice President, CSO, and Co-founder of Trophogen, Inc. discovering and developing biosuperior drugs for profit.
